# Dual Inhibition of MEK and PI3K Pathway in KRAS and BRAF Mutated Colorectal Cancers

**DOI:** 10.3390/ijms160922976

**Published:** 2015-09-23

**Authors:** Sally Temraz, Deborah Mukherji, Ali Shamseddine

**Affiliations:** Department of Internal Medicine, Hematology/Oncology Division, American University of Beirut Medical Center, Beirut 110 72020, Lebanon; E-Mails: dm25@aub.edu.lb (D.M.); as04@aub.edu.lb (A.S.)

**Keywords:** colorectal cancer, drug resistance, phosphatidylinositol 3-kinase, mitogen-activated protein kinase, MEK

## Abstract

Colorectal cancer (CRC) is a heterogeneous disease with multiple underlying causative genetic mutations. Genetic mutations in the phosphatidylinositol-3 kinase (PI3K) and the mitogen activated protein kinase (MAPK) pathways are frequently implicated in CRC. Targeting the downstream substrate MEK in these mutated tumors stands out as a potential target in CRC. Several selective inhibitors of MEK have entered clinical trial evaluation; however, clinical activity with single MEK inhibitors has been rarely observed and acquired resistance seems to be inevitable. Amplification of the driving oncogene KRAS(13D), which increases signaling through the ERK1/2 pathway, upregulation of the noncanonical wingless/calcium signaling pathway (Wnt), and coexisting PIK3CA mutations have all been implicated with resistance against MEK inhibitor therapy in KRAS mutated CRC. The Wnt pathway and amplification of the oncogene have also been associated with resistance to MEK inhibitors in CRCs harboring BRAF mutations. Thus, dual targeted inhibition of MEK and PI3K pathway effectors (mTOR, PI3K, AKT, IGF-1R or PI3K/mTOR inhibitors) presents a potential strategy to overcome resistance to MEK inhibitor therapy. Many clinical trials are underway to evaluate multiple combinations of these pathway inhibitors in solid tumors.

## 1. Introduction

Colorectal cancer (CRC) is a heterogeneous disease with multiple underlying causative genetic mutations. Activating genetic mutations in the phosphatidylinositol-3 kinase (PI3K) and the mitogen activated protein kinase (MAPK) pathways have been implicated in CRC. Insulin-like growth factor-1 receptor (IGF-1R) is a key activator of these two pro-survival signaling pathways [1]. In the PI3K pathway, activated receptor tyrosine kinases (RTK) cause phosphorylation of PI3K. Class 1 PI3K family members that convert phosphatidylinositol 4,5-bisphosphate (PIP2) to phosphatidylinositol 3,4,5-trisphosphate (PIP3) thereby activating AKT via two crucial phosphorylation events at threonine 308 catalyzed by phosphoinositide-dependent kinase-1 (PDK1) and at serine 473 catalyzed by mammalian target of rapamycin complex 2 (mTORC2) [2]. Phosphatase and tensin homolog (PTEN) reverses this process by dephosphorylating PIP3 to PIP2. Genetic anomalies of PI3K pathway components, such as PTEN loss, PI3K amplification/mutation, AKT mutation or RTK activation, result in activation of this pathway leading to tumorigensis [3]. Mutations in the PIK3CA gene, encoding the p110a catalytic subunit of class I PI3Ks are found in around 15% of CRCs [4].

The MAPK intracellular signaling cascade comprises RAS, RAF, MAPK extracellular signal-regulated kinase (MEK) and extracellular signal-regulated kinase (ERK1/2). The pathway is commonly activated through activating mutations of RAF or RAS. Activated MEK phosphorylates its only substrate ERK1/2 leading to dimerization, nuclear translocation and induction of target genes [5,6]. Mutations in the KRAS gene are the most common in CRCs comprising approximately 35%–45% [4]. Other mutations in this pathway, including NRAS and BRAF constitute approximately 4% and 8% of CRCs, respectively [4]. Thus, targeting the downstream substrate MEK in KRAS, BRAF or NRAS mutated tumors stands out as a potential target in CRC. Several selective inhibitors of MEK have entered clinical trial evaluation [7]; however, clinical activity with single MEK inhibitors has been rarely observed [8,9,10]. The first generation MEK inhibitor, CI-1040, was assessed in a phase II trial involving four tumor types. Generally, CI-1040 was well tolerated but failed to show sufficient antitumor activity to warrant further development [10]. In addition, the second-generation MEK1/2 inhibitor, Selumetinib (AZD6244), has not performed well in unselected patients with metastatic CRC. Selumetinib showed similar efficacy to capecitabine in terms of the number of patients with a disease progression event and progression-free survival [11]. Resistance to MEK inhibitors is largely due to compensatory up-regulation and crosstalk within the MAPK and PI3K pathways [12,13,14,15]. Identification of potential biomarkers for sensitivity or resistance to these agents is urgently needed. Moreover, with both of these pathways active in CRCs, targeting both pathways with small inhibitor molecules has shown greater clinical efficacy than single agents alone. With the increasing use of tumor sequencing for the purposes of treatment selection and identifying patients who may be eligible for clinical trials of agents targeting specific pathways, the molecular stratification of colorectal cancer is becoming a reality. Patients with KRAS/BRAF or NRAS mutated tumors are known to be resistant to monoclonal antibodies targeting the epidermal growth factor (EGFR), therefore options for palliative treatment are limited to cytotoxic chemotherapy, antti-angiogenic agents and most recently the multi-targeted tyrosine kinase inhibitor regorafenib that has been approved for use in patients refractory to these therapies in the third-line [16]. There is an urgent clinical need for the development of novel therapeutics that may be able to prolong survival in patients with RAS/BRAF mutated tumors.

## 2. Cross-Talk between MEK and PI3K Pathway

**Figure 1:**
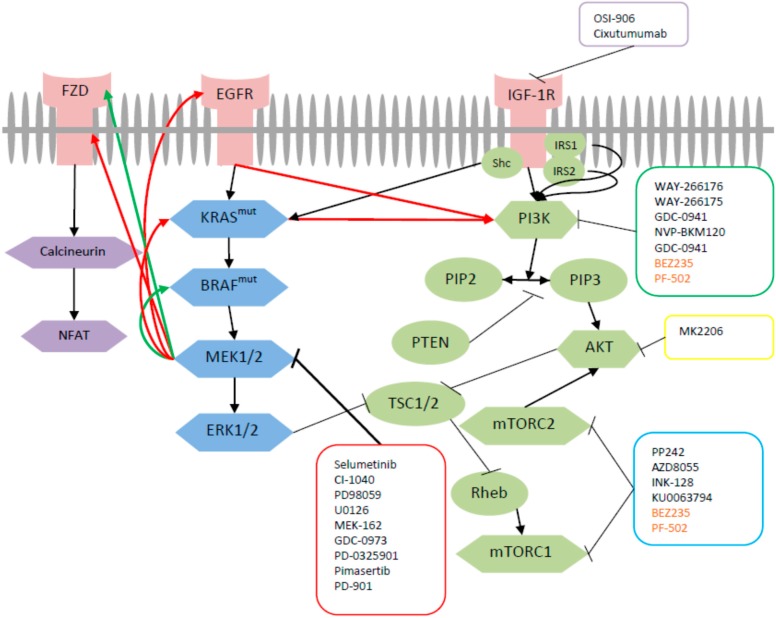
Cross talk between MAPK, PI3K and Wnt pathway in CRC. Upon MEK inhibition with one of the MEK inhibitors (shown in red box), KRAS mutated (Red lines) and BRAF mutated (Green lines) CRCs activate parallel pathways that incur resistance to MEK inhibition. Dual targeted inhibition of MEK with mTOR (shown in blue box), PI3K (shown in green box), AKT (shown in yellow box), IGF-1R (shown in purple box) or PI3K/mTOR (shown in orange) inhibitors has been studied to overcome this resistance. Regular arrow: activates; Arrow ending with a straight line: inhibits.

The frequent perturbation of the MAPK and PI3K signaling pathways in CRC make them promising candidates for the development of molecularly targeted agents. RAS and RAF gene mutations have been considered potential markers of sensitivity to MEK inhibition largely due to the constitutive activation of MEK seen in BRAF mutant cancer cells and to a lesser extent in KRAS mutant cells [17,18]. Thus, MEK has become a potential target for therapy given that mutation in these kinases has been identified as a negative predictor of benefit from EGFR inhibitor therapy [19,20,21]. However, acquired resistance after MEK inhibition seems inevitable. In KRAS mutated CRC cells, multiple mechanisms to resistance against MEK inhibition have been reported “Figure 1”. Amplification of the driving oncogene KRAS(13D) has been shown to drive acquired resistance to MEK1/2 inhibitors by increasing signaling through the ERK1/2 pathway [22,23]. Increased abundance of the oncogenic driver in response to prolonged drug treatment results in increased flux through the ERK pathway and restoration of ERK activity above the threshold required for cell growth [13]. The up-regulation of KRAS(13D) leads to activation of multiple KRAS effector pathways, thus highlighting the therapeutic challenge posed by KRAS mutations [22]. Upregulation of the noncanonical wingless/calcium signaling pathway (Wnt) signaling pathway is another mechanism by which KRAS mutated CRC cell lines showed resistance to selumetinib [24]. Coexisting mutations of the PIK3CA, which occur in about 8%–9% of CRC cases [25,26], have also been implicated in the resistance of KRAS mutated CRC cells to MEK inhibition [27,28]. In KRAS mutant tumors, PIK3CA mutation restores cyclin D1 expression and G(1)-S cell cycle progression so that they are no longer dependent on KRAS and MEK/ERK signaling [27]. Among RAF/RAS mutant lines, co-occurring PIK3CA/PTEN mutations conferred a cytostatic response instead of a cytotoxic response for colon cancer cells [28]. In another report, activating mutations in PIK3CA reduced the sensitivity to MEK inhibition, whereas PTEN mutations seemed to cause complete resistance. In addition, dual inhibition of the PI3K/AKT and RAF/MEK/ERK pathways also seems to be required for complete inhibition of the downstream mTOR effector pathway [29]. CRC cell lines that were resistant to selumetinib exhibited low or no ERK1/2 activation or exhibited coincident activation of ERK1/2 and protein kinase B (PKB), the latter indicative of activation of the PI3K pathway. Cells that were sensitive arrested in G(1) and/or underwent apoptosis and the presence of BRAF or KRAS mutation was not sufficient to predict either fate; however, CRC cells that did not harbor any mutation tended to be resistant [30]. Another study found that MEK inhibition resulted in activation of PI3K/AKT from the hyperactivation of ERBB3 as a result of the loss of an inhibitory threonine phosphorylation in the conserved juxtamembrane domains of EGFR and HER2. Mutation of this amino acid led to increased ERBB receptor activation and upregulation of the ERBB3/PI3K/AKT signaling pathway, which was no longer responsive to MEK inhibition [14]. Consecutive patients with diverse tumor types and PIK3CA mutation were treated whenever possible with agents targeting the PI3K/AKT/mTOR pathway. Of the 17 patients with PIK3CA mutations, 6 (35%) had simultaneous KRAS or BRAF mutations (colorectal, *n* = 4; ovarian, *n* = 2). Colorectal cancer patients with PIK3CA and KRAS mutations did not respond to therapy [31]. These data suggest that tumors with both KRAS and phosphoinositide 3-kinase mutations are unlikely to respond to the inhibition of the MEK pathway alone or the PI3K pathway alone but will require effective inhibition of both MEK and PI3K/AKT pathway signaling.

Tumor cells harboring the BRAF600E mutation have shown similar resistance to MEK1/2 inhibitors “Figure 1” by increasing signaling through the ERK1/2 pathway due to amplification of the oncogene [13,22]. For patients with BRAF mutant tumors, the results suggest that the addition of a RAF inhibitor to a MEK inhibitor may delay or overcome drug resistance [13]. Recently, the Wnt pathway was identified as a potential mediator of resistance to the MEK1/2 inhibitor selumetinib in tumors harboring BRAF mutation [32]. Concomitant use of selumetinib and cyclosporine, inhibited tumor growth and caused tumor regression in patient derived tumor xenografts [32].

## 3. Pre-Clinical Data on Dual Targeted Inhibition with MEK

### 3.1. Combined Inhibition of MEK and mTOR

Zhang *et al.* [33] assessed the combination effect of the MEK inhibitor PD98059 and mTOR inhibitor rapamycin on CRC cell lines. They found that combination treatment with PD98059 and rapamycin suppressed the proliferation of CRC cells, induced apoptosis, arrested cell cycle and reduced the incidence and volume of CRC in mice. They also demonstrated that combined administration of these two drugs was able to inhibit the phophorylation of mTOR and MEK signaling pathways and were significantly more effective than single agent treatment [33]. mTOR consists of two functionally distinct complexes, mTORC1 and mTORC2. mTORC1 has been shown to regulate cell growth by controlling mRNA translation initiation and to regulate ribosome biogenesis, autophagy and lipid biosynthesis [34]. mTORC2 on the other hand is involved in cell survival and proliferation through phosphorylation of AKT and protein kinase C [35]. mTORC1 has been shown to be sensitive to acute exposure to rapamycin while mTORC2 was not. Thus, ATP competitive inhibitors that target both mTOR complexes have been developed. Blaser *et al.* demonstrated that PP242, an ATP competitive inhibitor of mTORC1 and mTORC2, reduced the growth, proliferation and survival of colon cells more efficiently than rapamycin [36]. Moreover, the efficacy of ATP-competitive inhibitors of mTOR was enhanced by U0126, a MEK inhibitor. The authors also observed that ATP-competitive inhibitors of mTOR exhibited anticancer effects on both PIK3CA mutated as well as on PIK3CA wild type colon cancer cells [36]. Holt *et al.* also investigated the efficacy of AZD8055, a potent specific inhibitor of mTORC1 and mTORC2, in combination with selumetinib in human tumor non-small cell lung cancer (NSCLC) and CRC cell-derived xenograft and primary patient explants models [37]. Their results showed that combined use of AZD8055 and selumetinib enhanced antitumor activity, inhibited PI3K and MAPK pathways and induced apoptosis. Recently, Li *et al.* reported on the preclinical efficacy of INK-128, a novel ATP-competitive inhibitor of mTORC1 and mTORC2 [38]. INK-128 was capable of inhibiting CRC cell growth and survival and induced both apoptotic and non-apoptotic cancer cell death. INK-128 did not activate ERK and the use of the MEK inhibitor MEK-162 enhanced INK-128 cytotoxicity and inhibited Ht-29 xenograft growth in mice. Thus, combined use of MEK and mTOR inhibitors poses as a potential strategy in targeting not only tumors harboring KRAS mutation but also in those harboring PI3KCA mutation.

### 3.2. Combined MEK and PI3K Inhibition

Preclinical data suggest that combined MEK and PI3K inhibition will be required to enhance and prolong the anti-tumor activity of MEK inhibitors in KRAS-mutant colon cancers with alterations in the PI3K pathway. Yu *et al.* found that the HCT116 cell line, which harbors mutations in both KRAS and PIK3CA, was resistant to the PI3K inhibitors WAY-266176 and WAY-266175; however, combined therapy with the MEK inhibitors CI-1040 or UO126 led to growth suppression and apoptosis [39]. Data revealed that the down-regulation of PI3K re-sensitizes cell harboring mutations in KRAS and PIK3CA to MEK inhibition while re-expression of mutant PI3KCA allele in MEK inhibitor-resistant cells restores MEK pathway sensitivity [27,29]. Thus, the dual inhibition of both pathways seems to be required for complete inhibition and the induction of cell death. Hoeflich *et al.* further demonstrated that combined treatment with the MEK inhibitor GDC-0973 and PI3K inhibitor GDC-0941 results in apoptosis and growth inhibition of BRAF and KRAS mutated cell lines and human xenograft tumor models [40]. Recently, Roper *et al.* have also demonstrated that combined PI3K and MEK inhibition with NVP-BKM120 and PD-0325901 induced tumor regression in a mouse model of PIK3CA wild-type and KRAS mutant colorectal cancer [41]. These data indicate that dual MEK and PI3K inhibition is a valid strategy in both PIK3CA wild-type and PIK3CA mutated CRCs with KRAS and/or BRAF mutations and that dual pathway inhibition may be more effective than inhibiting either single pathway alone.

### 3.3. Combined MEK and P13K/mTOR Inhibition

mTOR inhibition has been correlated with activation of MAPK pathway through PI3K’s activation of RAS [42]. Thus, multiple preclinical studies have evaluated the efficacy of MEK inhibition combined with both PI3K and mTOR inhibitors. Martinelli *et al*. revealed that the selective MEK1/2 inhibitor pimasertib combined with the dual PI3K/mTOR inhibitor BEZ235 or with sorafenib caused significant tumor growth delays and increased survival in mice as compared to single agent treatment thereby suggesting that dual blockade of MAPK and PI3K pathways could overcome intrinsic resistance to MEK inhibition [43]. Migliardi *et al.* reported on an analysis of forty different patient-derived murine xenografts of metastatic CRC [44]. They found that the combination of selumetinib and BEZ235 achieved greater rates of disease stabilization than monotherapy (70% *vs.* 27.5% for selumetinib alone and 42.5% for BEZ235 alone). However, no substantial tumor regression was observed for combined therapy suggesting that this combination could be useful only in retarding disease progression. Similarly, Pitt *et al.* demonstrated synergistic anti-proliferative activity with the combination of PI3K/mTOR inhibitor PF-502 and the MEK1/2 inhibitor PD-901 [45]. Combination treatment demonstrated enhanced reduction in tumor growth against CRC cell line and patient-derived tumor xenograft models as compared to either single agent regardless of KRAS or PI3K mutational status. Recently, E *et al.* [46] also reported their results on the use of BEZ235 with selumetinib in HCT116 CRC cells harboring both KRAS and PIk3CA mutations. The combination treatment markedly enhanced their antitumor effects than either single agent alone. The authors also reported a reduction in the phosphorylation level of ERK1/2 and AKT and in the expression of VEGF and matrix metallopeptidase-9 in tumor tissue [46].

When comparing dual PI3K/mTOR inhibition with MEK inhibition against PI3K and MEK inhibition, Haagensen *et al.* found that the pan-PI3K inhibitor GDC-0941 demonstrated greater synergy than the dual PI3K/mTOR inhibitor BEZ235 when combined with the MEK inhibitors selumetinib and PD0325901 in BRAF/PIK3CA-double-mutant HT29 and KRAS/PIK3CA-double-mutant HCT116 CRC cell lines [47]. BEZ235 resulted in marked inhibition of 4EBP1 phosphorylation alone and in combination, whereas there was minimal inhibition with GDC-0941, suggesting that the dual mTOR/PI3K inhibitory activity of BEZ235 may underlie its ability to inhibit 4EBP1 phosphorylation. The addition of the mTORC1/2 inhibitor KU0063794 to GDC-0941 and selumetinib also compromised the synergy achieved between GDC-0941 and selumetinib and was likely due to mTOR inhibition with the addition of KU0063794. The results suggest that the combination of specific PI3K inhibitors, rather than dual mTOR/PI3K inhibitors, with MEK inhibitors results in greater synergy. Thus, these preclinical data suggest that MEK and PI3K/mTOR inhibition may delay tumor growth but may not affect tumor size regression and is likely to be inferior to MEK and PI3K inhibition.

### 3.4. Combined MEK and AKT Inhibition

AKT upregulation occurs in approximately 60% of CRC cases which renders AKT a potential target for inhibition [48]. Halilovic *et al.* [27] observed increased antitumor activity with combined MEK and AKT inhibition in KRAS/PIK3CA-double-mutant HCT15. Mutant HCT15 tumor xenografts were treated with the MEK inhibitor PD0325901 and AKTi-1/2 alone or in combination. Treatment with either agent alone had no significant effect on tumor growth; however, combined treatment abrogated the growth of tumor xenografts [27].

### 3.5. Combined Inhibition of MEK and IGFR

IGF-1R is a key regulator of both PI3K and MAPK pathways; however, trials of single agent inhibitors of IGF-1R have shown only modest clinical responses [49]. Previous experimentation with OSI-906, an (IGF1R)/insulin receptor (IR) tyrosine kinase inhibitor (TKI), revealed resistance to OSI-906 that was coupled with simultaneous activation of the MAPK pathway in CRC cell lines [50]. Thus, Flanigan *et al.* [51] tested the efficacy of OSI-906 in combination with selumetinib and U0126. The combination of OSI-906 and U0126 revealed synergistic effects in 11 out of 13 CRC cell lines. In addition, *in vivo* xenograft studies with OSI-906 and selumetinib showed synergistic effects that resulted in apoptosis or cell cycle arrest [51].

## 4. Clinical Trials on Dual Targeted Inhibition with MEK

Data from patients treated with phase I study drugs targeting the PI3K and/or MAPK pathways revealed that CRC patients harboring mutations in both pathways had tumor regression ranging between 2% and 64% when treated with PI3K and MAPK inhibitors. Dual pathway inhibition may potentially exhibit greater efficacy than single targeted therapy at the expense of greater toxicity. In this study, the most frequent drug-related grade >III adverse events were liver transaminase elevations, mucositis and skin rash [52]. Other clinical trials targeting MEK and one or more effectors of the PI3K pathway are underway and whose results are eagerly awaited particularly concerning patients with KRAS or concomitant KRAS and PIK3CA mutations “Table 1”.

Cixutumumab is a recombinant human IgG1/monoclonal antibody that blocks interaction of IGF-1R and ligands IGF-1 and IGF-2 and thus leads to degradation of IGF-1R. Combination of cixutumumab and selumetinib were evaluated recently in an open label, phase I dose-escalation clinical trial in patients with advanced solid tumors. Wilky *et al*. reported the combination to be well tolerated at maximum doses of 50 mg twice daily for selumetinib and 12 mg/kg every two weeks for cixutumumab. Six patients out of 30 achieved time to progression of >6 months, including patients with thyroid carcinoma, colorectal carcinoma and basal cell carcinoma and two patients achieved partial responses [53]. The results revealed preliminary evidence of clinical benefit and pharmacodynamic evidence of targeted inhibition with IGF-1R and MEK inhibition and which deserve further evaluation in clinical trials.

In a recent biomarker phase 2 trial, Do *et al*. [54] reported the results of combined inhibition with selumetinib and MK-2206, an allosteric inhibitor of the three human isoforms of AKT. Of the 21 patients with advanced CRC enrolled, none reported an objective response regardless of KRAS mutational status. The desired level of target inhibition of pERK and pAKT levels in paired tumor biopsies was not achieved. Common toxicities were gastrointestinal, hepatic, dermatologic, and hematologic. These toxicities hindered the possibility of dose escalation to achieve levels needed for clinical activity [54]. Based on these results, the authors did not recommend the further evaluation of this combination until appropriate dosing is established in these tumors. One phase I clinical trial in advanced solid tumors targeting both AKT and MEK with GDC-0068 and GDC-0973 “Table 1” is underway and which will aim to evaluate the safety and tolerability of this combination. Until those results are available, current evidence does not support the use of this combination in metastatic CRC patients.

**Table 1:** Ongoing clinical trials on MEK and PI3K pathway inhibitors in colorectal cancer.

Trial	MEK Inhibitor	PI3K Inhibitor	Phase	Population
NCT01363232	MEK162	BKM120	Phase I	Patients with advanced solid cancers (colorectal cancer, triple-negative breast cancer, pancreatic cancer, and other cancers harboring KRAS, BRAF and NRAS mutations)
NCT01392521	BAY86-9766	BAY80-6946	Phase Ib	Patients with advanced solid cancers
NCT01449058	MEK162	BYL719	Phase II	Patients with advanced solid cancers (colorectal cancer, esophageal cancer, pancreatic cancer, non-small cell lung cancer, and other advanced solid tumors harboring RAS or BRAF mutations)
**Trial**	**MEK Inhibitor**	**PI3K/mTOR Inhibitor**	**Phase**	**Population**
NCT00996892	GDC-0973	Pictilisb (GDC-0941)	Phase I	Patients with advanced solid cancers
NCT01337765	MEK162	BEZ235	Phase 1	Patients with advanced solid cancers (colorectal cancer, triple-negative breast cancer, pancreatic cancer, malignant melanoma, non-small cell lung cancer, and other cancers harboring KRAS, BRAF and NRAS mutation)
NCT01390818	Pimasertib (MSC1936369B)	SAR245409	Phase 1	Patients with advanced solid cancers (colorectal cancer, pancreatic cancer, thyroid cancer, non-small cell lung cancer, renal cancer, breast cancer, melanoma, ovarian cancer) and any cancer diagnosed with aberrations in one or more the following genes: PTEN, BRAF, KRAS, NRAS, PI3KCA, ErbB1, ErbB2)
NCT01347866	PD0325901	PF-05212384	Phase 1	Patients with advanced solid cancers harboring KRAS or BRAF mutation and patients with KRAS mutation with no more than one prior systemic therapy regimen
**Trial**	**MEK Inhibitor**	**AKT Inhibitor**	**Phase**	**Population**
NCT01562275	GDC-0973	GDC-0068	Phase 1	Patients with locally advanced or metastatic solid tumors

## 5. Conclusions

Currently, no targeted KRAS inhibitors exist for KRAS mutated CRC patients. Thus, MEK inhibitors have been studied in this patient population and to a lesser extent in patients harboring BRAF mutated tumors. Regardless of initial response to MEK inhibition, tumors eminently become resistant as a result of various alternative signaling pathways that induce cell proliferation and survival. Thus multiple pathway inhibition has become the focus of treatment to overcome this resistance seen with MEK inhibition “Figure 1”.

Preclinical data thus far support dual targeted inhibition of MEK and one or more of the PI3K pathway effectors in metastatic CRC, which was superior to single agent alone. Many clinical trials are underway to evaluate multiple combinations of these pathway inhibitors in solid tumors. But until the results of these are available and the safety and tolerability levels of these targeted inhibitors are established, KRAS and BRAF mutated CRCs remain to have limited treatment options. Preclinical data also suggest that combined MEK and PI3K inhibition is more effective than MEK and PI3K/mTOR inhibition, which needs to be further evaluated. Co-targeted inhibition of IGF-1R and MEK has also shown potential in a recent phase I trial and will require further clinical evaluation in the future. Preclinical data have also identified the Wnt pathway as being activated in KRAS and BRAF mutated tumors treated with MEK inhibitors. The combination of a Wnt pathway inhibitor such as cyclosporine and a MEK1/2 inhibitor needs to be clinically evaluated. Future clinical trials with appropriate molecular stratification and biomarker development have the potential to improve outcomes for patients suffering from advanced colorectal cancer for whom current palliative treatment options are limited.

## References

[1] LeRoith D., Roberts C.T. (2003). The insulin-like growth factor system and cancer. Cancer Lett..

[2] Sarbassov D.D., Guertin D.A., Ali S.M., Sabatini D.M. (2005). Phosphorylation and regulation of Akt/PKB by the rictor-mTOR complex. Science.

[3] Yuan T.L., Cantley L.C. (2008). PI3K pathway alterations in cancer: Variations on a theme. Oncogene.

[4] De Roock W., de Vriendt V., Normanno N., Ciardiello F., Tejpar S. (2011). KRAS, BRAF, PIK3CA, and PTEN mutations: Implications for targeted therapies in metastatic colorectal cancer. Lancet Oncol..

[5] Chang L., Karin M. (2001). Mammalian map kinase signalling cascades. Nature.

[6] Khokhlatchev A.V., Canagarajah B., Wilsbacher J., Robinson M., Atkinson M., Goldsmith E., Cobb M.H. (1998). Phosphorylation of the MAP kinase ERK2 promotes its homodimerization and nuclear translocation. Cell.

[7] Fremin C., Meloche S. (2010). From basic research to clinical development of MEK1/2 inhibitors for cancer therapy. J. Hematol. Oncol..

[8] Banerji U., Camidge D.R., Verheul H.M., Agarwal R., Sarker D., Kaye S.B., Desar I.M., Timmer-Bonte J.N., Eckhardt S.G., Lewis K.D. (2010). The first-in-human study of the hydrogen sulfate (Hyd-sulfate) capsule of the MEK1/2 inhibitor AZD6244 (ARRY-142886): A phase I open-label multicenter trial in patients with advanced cancer. Clin. Cancer Res..

[9] Lorusso P.M., Adjei A.A., Varterasian M., Gadgeel S., Reid J., Mitchell D.Y., Hanson L., DeLuca P., Bruzek L., Piens J. (2005). Phase I and pharmacodynamic study of the oral MEK inhibitor CI-1040 in patients with advanced malignancies. J. Clin. Oncol..

[10] Rinehart J., Adjei A.A., Lorusso P.M., Waterhouse D., Hecht J.R., Natale R.B., Hamid O., Varterasian M., Asbury P., Kaldjian E.P. (2004). Multicenter phase II study of the oral MEK inhibitor, CI-1040, in patients with advanced non-small-cell lung, breast, colon, and pancreatic cancer. J. Clin. Oncol..

[11] Bennouna J., Lang I., Valladares-Ayerbes M., Boer K., Adenis A., Escudero P., Kim T.Y., Pover G.M., Morris C.D., Douillard J.Y. (2011). A phase II, open-label, randomised study to assess the efficacy and safety of the MEK1/2 inhibitor AZD6244 (ARRY-142886) *versus* capecitabine monotherapy in patients with colorectal cancer who have failed one or two prior chemotherapeutic regimens. Investig. New Drug..

[12] O’Reilly K.E., Rojo F., She Q.B., Solit D., Mills G.B., Smith D., Lane H., Hofmann F., Hicklin D.J., Ludwig D.L. (2006). mTOR inhibition induces upstream receptor tyrosine kinase signaling and activates Akt. Cancer Res..

[13] Poulikakos P.I., Solit D.B. (2011). Resistance to MEK inhibitors: Should we co-target upstream?. Sci. Signal..

[14] Turke A.B., Song Y., Costa C., Cook R., Arteaga C.L., Asara J.M., Engelman J.A. (2012). MEK inhibition leads to PI3K/Akt activation by relieving a negative feedback on ERBB receptors. Cancer Res..

[15] Britten C.D. (2013). PI3K and MEK inhibitor combinations: Examining the evidence in selected tumor types. Cancer Chemother. Pharm..

[16] Temraz S., Mukherji D., Shamseddine A. (2014). Sequencing of treatment in metastatic colorectal cancer: Where to fit the target. World J. Gastroenterol..

[17] Solit D.B., Garraway L.A., Pratilas C.A., Sawai A., Getz G., Basso A., Ye Q., Lobo J.M., She Y., Osman I. (2006). Braf mutation predicts sensitivity to MEK inhibition. Nature.

[18] Davies B.R., Logie A., McKay J.S., Martin P., Steele S., Jenkins R., Cockerill M., Cartlidge S., Smith P.D. (2007). AZD6244 (ARRY-142886), a potent inhibitor of mitogen-activated protein kinase/extracellular signal-regulated kinase kinase 1/2 kinases: Mechanism of action *in vivo*, pharmacokinetic/pharmacodynamic relationship, and potential for combination in preclinical models. Mol. Cancer Ther..

[19] Cui D., Cao D., Yang Y., Qiu M., Huang Y., Yi C. (2014). Effect of BRAF V600E mutation on tumor response of anti-EGFR monoclonal antibodies for first-line metastatic colorectal cancer treatment: A meta-analysis of randomized studies. Mol. Biol. Rep..

[20] Sorich M.J., Wiese M.D., Rowland A., Kichenadasse G., McKinnon R.A., Karapetis C.S. (2014). Extended RAS mutations and anti-EGFR monoclonal antibody survival benefit in metastatic colorectal cancer: A meta-analysis of randomized, controlled trials. Ann. Oncol..

[21] Li W., Shi Q., Wang W., Liu J., Ren J., Li Q., Hou F. (2014). KRAS status and resistance to epidermal growth factor receptor tyrosine-kinase inhibitor treatment in patients with metastatic colorectal cancer: A meta-analysis. Colorectal Dis..

[22] Little A.S., Balmanno K., Sale M.J., Newman S., Dry J.R., Hampson M., Edwards P.A., Smith P.D., Cook S.J. (2011). Amplification of the driving oncogene, KRAS or BRAF, underpins acquired resistance to MEK1/2 inhibitors in colorectal cancer cells. Sci. Signal..

[23] Wang Y., van Becelaere K., Jiang P., Przybranowski S., Omer C., Sebolt-Leopold J. (2005). A role for K-ras in conferring resistance to the MEK inhibitor, CI-1040. Neoplasia.

[24] Tentler J.J., Nallapareddy S., Tan A.C., Spreafico A., Pitts T.M., Morelli M.P., Selby H.M., Kachaeva M.I., Flanigan S.A., Kulikowski G.N. (2010). Identification of predictive markers of response to the MEK1/2 inhibitor selumetinib (AZD6244) in K-ras-mutated colorectal cancer. Mol. Cancer Ther..

[25] Nosho K., Kawasaki T., Ohnishi M., Suemoto Y., Kirkner G.J., Zepf D., Yan L., Longtine J.A., Fuchs C.S., Ogino S. (2008). PIK3CA mutation in colorectal cancer: Relationship with genetic and epigenetic alterations. Neoplasia.

[26] De Roock W., Claes B., Bernasconi D., de Schutter J., Biesmans B., Fountzilas G., Kalogeras K.T., Kotoula V., Papamichael D., Laurent-Puig P. (2010). Effects of KRAS, BRAF, NRAS, and PIK3CA mutations on the efficacy of cetuximab plus chemotherapy in chemotherapy-refractory metastatic colorectal cancer: A retrospective consortium analysis. Lancet Oncol..

[27] Halilovic E., She Q.B., Ye Q., Pagliarini R., Sellers W.R., Solit D.B., Rosen N. (2010). PIK3CA mutation uncouples tumor growth and cyclin D1 regulation from MEK/ERK and mutant KRAS signaling. Cancer Res..

[28] Jing J., Greshock J., Holbrook J.D., Gilmartin A., Zhang X., McNeil E., Conway T., Moy C., Laquerre S., Bachman K. (2012). Comprehensive predictive biomarker analysis for MEK inhibitor GSK1120212. Mol. Cancer Ther..

[29] Wee S., Jagani Z., Xiang K.X., Loo A., Dorsch M., Yao Y.M., Sellers W.R., Lengauer C., Stegmeier F. (2009). PI3K pathway activation mediates resistance to MEK inhibitors in KRAS mutant cancers. Cancer Res..

[30] Balmanno K., Chell S.D., Gillings A.S., Hayat S., Cook S.J. (2009). Intrinsic resistance to the MEK1/2 inhibitor AZD6244 (ARRY-142886) is associated with weak ERK1/2 signalling and/or strong PI3K signalling in colorectal cancer cell lines. Int. J. Cancer.

[31] Janku F., Tsimberidou A.M., Garrido-Laguna I., Wang X., Luthra R., Hong D.S., Naing A., Falchook G.S., Moroney J.W., Piha-Paul S.A. (2011). PIK3CA mutations in patients with advanced cancers treated with PI3K/AKT/mTOR axis inhibitors. Mol. Cancer Ther..

[32] Spreafico A., Tentler J.J., Pitts T.M., Tan A.C., Gregory M.A., Arcaroli J.J., Klauck P.J., McManus M.C., Hansen R.J., Kim J. (2013). Rational combination of a MEK inhibitor, selumetinib, and the Wnt/calcium pathway modulator, cyclosporin A, in preclinical models of colorectal cancer. Clin. Cancer Res..

[33] Zhang Y.J., Tian X.Q., Sun D.F., Zhao S.L., Xiong H., Fang J.Y. (2009). Combined inhibition of MEK and mTOR signaling inhibits initiation and progression of colorectal cancer. Cancer Investig..

[34] Ma X.M., Blenis J. (2009). Molecular mechanisms of mTOR-mediated translational control. Nat. Rev. Mol. Cell Biol..

[35] Ikenoue T., Inoki K., Yang Q., Zhou X., Guan K.L. (2008). Essential function of TORC2 in PKC and Akt turn motif phosphorylation, maturation and signalling. EMBO J..

[36] Blaser B., Waselle L., Dormond-Meuwly A., Dufour M., Roulin D., Demartines N., Dormond O. (2012). Antitumor activities of ATP-competitive inhibitors of mTOR in colon cancer cells. BMC Cancer.

[37] Holt S.V., Logie A., Davies B.R., Alferez D., Runswick S., Fenton S., Chresta C.M., Gu Y., Zhang J., Wu Y.L. (2012). Enhanced apoptosis and tumor growth suppression elicited by combination of MEK (selumetinib) and mTOR kinase inhibitors (AZD8055). Cancer Res..

[38] Li C., Cui J.F., Chen M.B., Liu C.Y., Liu F., Zhang Q.D., Zou J., Lu P.H. (2015). The preclinical evaluation of the dual mTORC1/2 inhibitor INK-128 as a potential anti-colorectal cancer agent. Cancer Biol. Ther..

[39] Yu K., Toral-Barza L., Shi C., Zhang W.G., Zask A. (2008). Response and determinants of cancer cell susceptibility to PI3K inhibitors: Combined targeting of PI3K and MEK1 as an effective anticancer strategy. Cancer Biol. Ther..

[40] Hoeflich K.P., Merchant M., Orr C., Chan J., Den Otter D., Berry L., Kasman I., Koeppen H., Rice K., Yang N.Y. (2012). Intermittent administration of MEK inhibitor GDC-0973 plus PI3K inhibitor GDC-0941 triggers robust apoptosis and tumor growth inhibition. Cancer Res..

[41] Roper J., Sinnamon M.J., Coffee E.M., Belmont P., Keung L., Georgeon-Richard L., Wang W.V., Faber A.C., Yun J., Yilmaz O.H. (2014). Combination PI3K/MEK inhibition promotes tumor apoptosis and regression in PIK3CA wild-type, KRAS mutant colorectal cancer. Cancer Lett..

[42] Carracedo A., Pandolfi P.P. (2008). The PTEN-PI3K pathway: Of feedbacks and cross-talks. Oncogene.

[43] Martinelli E., Troiani T., D’Aiuto E., Morgillo F., Vitagliano D., Capasso A., Costantino S., Ciuffreda L.P., Merolla F., Vecchione L. (2013). Antitumor activity of pimasertib, a selective MEK 1/2 inhibitor, in combination with PI3K/mTOR inhibitors or with multi-targeted kinase inhibitors in pimasertib-resistant human lung and colorectal cancer cells. Int. J. Cancer..

[44] Migliardi G., Sassi F., Torti D., Galimi F., Zanella E.R., Buscarino M., Ribero D., Muratore A., Massucco P., Pisacane A. (2012). Inhibition of MEK and PI3K/mTOR suppresses tumor growth but does not cause tumor regression in patient-derived xenografts of RAS-mutant colorectal carcinomas. Clin. Cancer Res..

[45] Pitts T.M., Newton T.P., Bradshaw-Pierce E.L., Addison R., Arcaroli J.J., Klauck P.J., Bagby S.M., Hyatt S.L., Purkey A., Tentler J.J. (2014). Dual pharmacological targeting of the map kinase and PI3K/mTOR pathway in preclinical models of colorectal cancer. PLoS ONE.

[46] E J., Xing J., Gong H., He J., Zhang W. (2015). Combine MEK inhibition with PI3K/mTOR inhibition exert inhibitory tumor growth effect on KRAS and PIK3CA mutation CRC xenografts due to reduced expression of vegf and matrix metallopeptidase-9. Tumor Biol..

[47] Haagensen E.J., Kyle S., Beale G.S., Maxwell R.J., Newell D.R. (2012). The synergistic interaction of MEK and PI3K inhibitors is modulated by mTOR inhibition. Br. J. Cancer.

[48] Roy H.K., Olusola B.F., Clemens D.L., Karolski W.J., Ratashak A., Lynch H.T., Smyrk T.C. (2002). AKT proto-oncogene overexpression is an early event during sporadic colon carcinogenesis. Carcinogenesis.

[49] Jin M., Petronella B.A., Cooke A., Kadalbajoo M., Siu K.W., Kleinberg A., May E.W., Gokhale P.C., Schulz R., Kahler J. (2013). Discovery of novel insulin-like growth factor-1 receptor inhibitors with unique time-dependent binding kinetics. ACS Med. Chem. Lett..

[50] Pitts T.M., Tan A.C., Kulikowski G.N., Tentler J.J., Brown A.M., Flanigan S.A., Leong S., Coldren C.D., Hirsch F.R., Varella-Garcia M. (2010). Development of an integrated genomic classifier for a novel agent in colorectal cancer: Approach to individualized therapy in early development. Clin. Cancer Res..

[51] Flanigan S.A., Pitts T.M., Newton T.P., Kulikowski G.N., Tan A.C., McManus M.C., Spreafico A., Kachaeva M.I., Selby H.M., Tentler J.J. (2013). Overcoming IGF1R/IR resistance through inhibition of MEK signaling in colorectal cancer models. Clin. Cancer Res..

[52] Shimizu T., Tolcher A.W., Papadopoulos K.P., Beeram M., Rasco D.W., Smith L.S., Gunn S., Smetzer L., Mays T.A., Kaiser B. (2012). The clinical effect of the dual-targeting strategy involving PI3K/AKT/mTOR and RAS/MEK/ERK pathways in patients with advanced cancer. Clin. Cancer Res..

[53] Wilky B.A., Rudek M.A., Ahmed S., Laheru D.A., Cosgrove D., Donehower R.C., Nelkin B., Ball D., Doyle L.A., Chen H. (2015). A phase I trial of vertical inhibition of IGF signalling using cixutumumab, an anti-IGF-1R antibody, and selumetinib, an MEK 1/2 inhibitor, in advanced solid tumours. Br. J. Cancer.

[54] Do K., Speranza G., Bishop R., Khin S., Rubinstein L., Kinders R.J., Datiles M., Eugeni M., Lam M.H., Doyle L.A. (2015). Biomarker-driven phase 2 study of MK-2206 and selumetinib (AZD6244, ARRY-142886) in patients with colorectal cancer. Investig. New Drug.

